# Gallstone Ileus following Endoscopic Stone Extraction

**DOI:** 10.1155/2014/271571

**Published:** 2014-09-28

**Authors:** Yoshiya Yamauchi, Noritaka Wakui, Yasutsugu Asai, Nobuhiro Dan, Yuki Takeda, Nobuo Ueki, Takahumi Otsuka, Nobuyuki Oba, Shuta Nisinakagawa, Tatsuya Kojima

**Affiliations:** Department of Internal Gastroenterology and Hepatology, Tokyo Rosai Hospital, 4-13-21 Omori-minami, Ota-ku, Tokyo 143-0013, Japan

## Abstract

An 85-year-old woman was an outpatient treated at Tokyo Rosai Hospital for cirrhosis caused by hepatitis B. She had previously been diagnosed as having common bile duct stones, for which she underwent endoscopic retrograde cholangiopancreatography (ERCP). However, as stone removal was unsuccessful, a plastic stent was placed after endoscopic sphincterotomy. In October 2012, the stent was replaced endoscopically because she developed cholangitis due to stent occlusion. Seven days later, we performed ERCP to treat recurring cholangitis. During the procedure, the stone was successfully removed by a balloon catheter when cleaning the common bile duct. The next day, the patient developed abdominal pain, abdominal distension, and nausea and was diagnosed as having gallstone ileus based on abdominal computed tomography (CT) and abdominal ultrasonography findings of an incarcerated stone in the terminal ileum. Although colonoscopy was performed after inserting an ileus tube, no stone was visible. Subsequent CT imaging verified the disappearance of the incarcerated stone from the ileum, suggesting that the stone had been evacuated naturally via the transanal route. Although it is extremely rare for gallstone ileus to develop as a complication of ERCP, physicians should be aware of gallstone ileus and follow patients carefully, especially after removing huge stones.

## 1. Introduction

Ileus is a disease that should be differentiated as a cause of abdominal pain and nausea in elderly individuals. Among the different types of ileus, gallstone ileus is a rare condition associated with bowel obstruction caused by a gallstone passing into the intestinal tract, and this disease accounts for 1–3% of cases of mechanical obstruction of the small intestine and 25% of cases of nonstrangulation ileus in individuals >65 years old [1, 2]. Gallstone ileus generally develops when gallstones pass into the intestinal tract through the fistula formed between the gallbladder and the gastrointestinal tract due to cholecystitis [2], and it is extremely rare to observe gallstone ileus after endoscopic retrograde cholangiopancreatography (ERCP) [1]. Here, we report a rare case of gallstone ileus that developed after endoscopic sphincterotomy (ES) and the successful endoscopic removal of a huge stone using a balloon catheter.

## 2. Case Report

**Figure 1:**
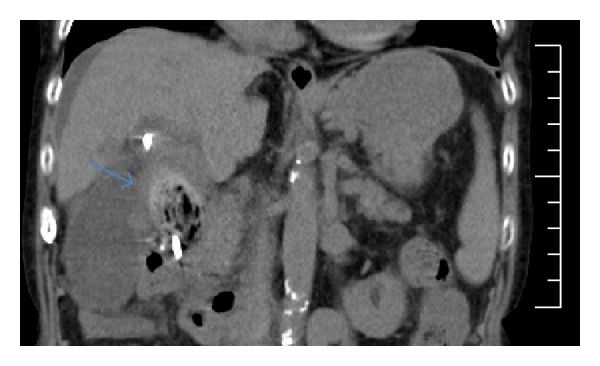
Abdominal computed tomographic (CT) image showing the common bile duct stone (arrow) accompanied by fat stranding in the surrounding adipose tissue on hospital day 47.

**Figure 2:**
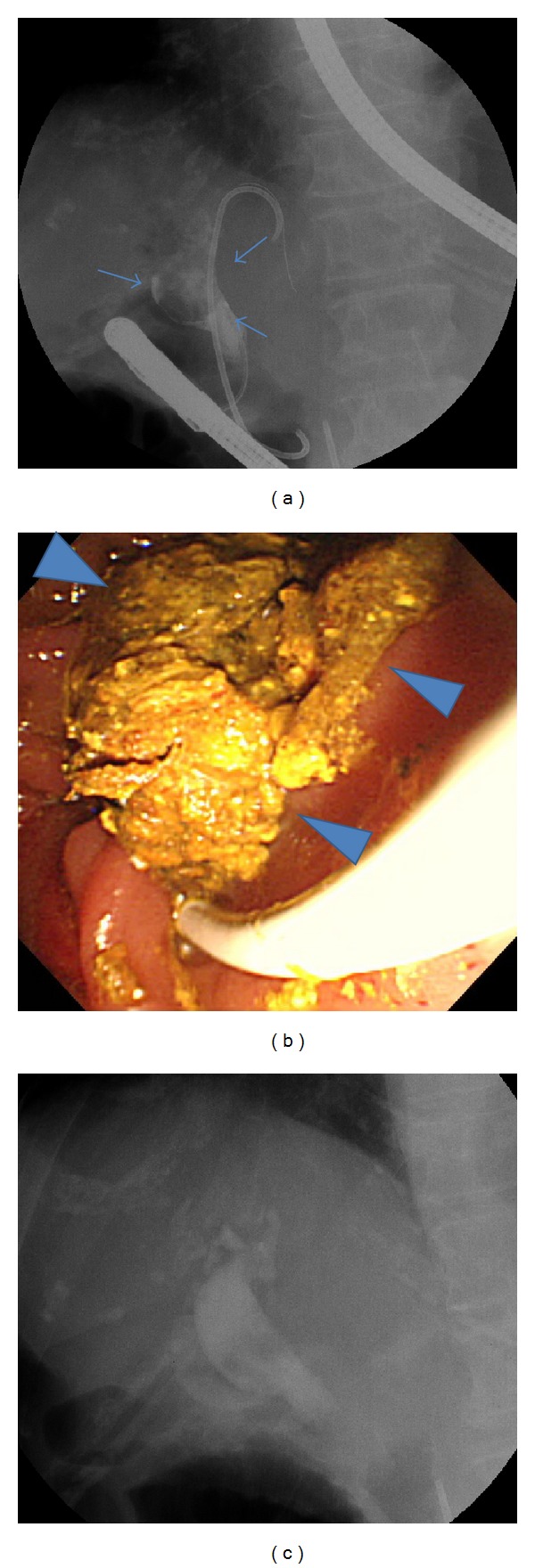
Endoscopic retrograde cholangiopancreatography (ERCP) images on hospital day 57. (a) Long-standing huge stone in the common bile duct (arrows). (b) A large amount of biliary sludge (arrowheads) and the stone, which had been difficult to remove, were washed out by a balloon catheter inserted to drain biliary sludge and infected bile (unable to photograph the stone). (c) Enhanced CT image showing no stone or biliary sludge remaining in the common bile duct after cleaning.

An 85-year-old woman, who had been treated for hepatitis B virus-related cirrhosis at Tokyo Rosai Hospital, was diagnosed as having common bile duct stones during a routine abdominal ultrasonographic examination in January 2009, for which she underwent ERCP in July 2009. However, because the Vater papilla was located inside a diverticulum in the third portion of the duodenum, the use of a stone crusher was difficult. Therefore, a double-pigtail plastic stent (Zimmon Biliary Stent, 4 cm, 7 Fr; Cook Japan, Tokyo, Japan) was placed to prevent post-ES cholecystitis, and the stent was replaced usually every 3 to 12 months. An interval was decided according to her general condition. In September 2012, the patient was admitted to the Department of Gastroenterological Medicine for the management of pain due to a lumbar compression fracture and hepatic encephalopathy. Hepatic encephalopathy improved after admission, and the patient was undergoing rehabilitation for lumbar compression fracture when she developed abdominal pain and fever on hospital day 47. Physical examination revealed yellowing of the palpebral conjunctiva and tenderness in the epigastric region. Hematological findings were a high white blood cell count, low level of hemoglobin, low platelet count, and reduced prothrombin time. The patient also had hypoproteinemia, renal dysfunction, and elevated levels of bilirubin, hepatic enzymes, and C-reactive protein (Table 1). Computed tomography (CT) revealed a stone in the common bile duct and depicts extensive fat inflammation surrounding the common bile duct in the surrounding adipose tissue (Figure 1). Based on these findings, the patient was diagnosed as having acute cholecystitis and underwent ERCP on hospital day 48 to replace her stent with a new plastic stent (biliary stent kit, 4 cm, 7 Fr, Cathex Co., Tokyo, Japan). Later, oral intake was resumed. On hospital day 55, however, the patient developed further episodes of fever and abdominal pain and had elevated levels of hepatobiliary enzymes. Two days later, ERCP was performed again. Because of the appearance of biliary sludge after stent removal, we inserted a balloon catheter (Extractor Pro XL 12/15 mm; Boston Scientific Japan, Tokyo, Japan) to drain the biliary sludge and infected bile in the common bile duct. During cleaning, the stone, which had been difficult to remove in previous surgeries, was passed into the duodenum. Thereafter, a large amount of biliary sludge was removed by cleaning the common bile duct several times. CT was performed to verify the stone removal before completing surgery (Figure 2).

**Figure 3:**
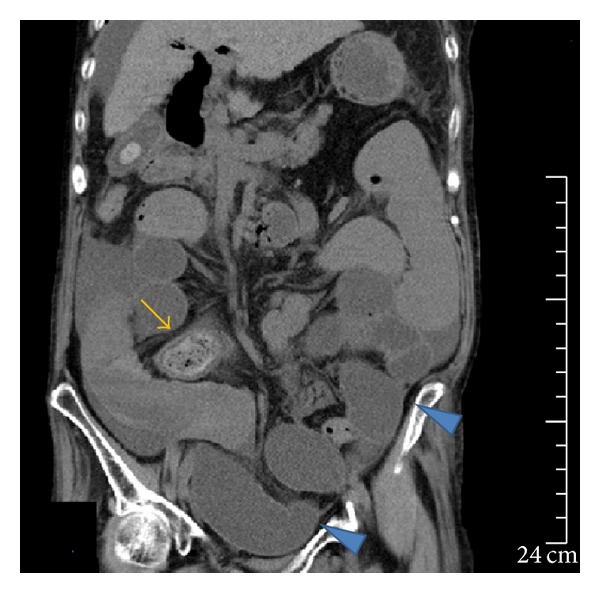
Abdominal CT image showing the stone (arrow) in the ileocecal region on hospital day 59. The stone was successfully passed into the duodenum in the previous ERCP. The image also shows distension of the small intestine proximal to the stone and the accumulation of intraluminal fluid (arrowheads), the latter of which had increased since CT imaging on hospital day 47.

**Figure 4:**
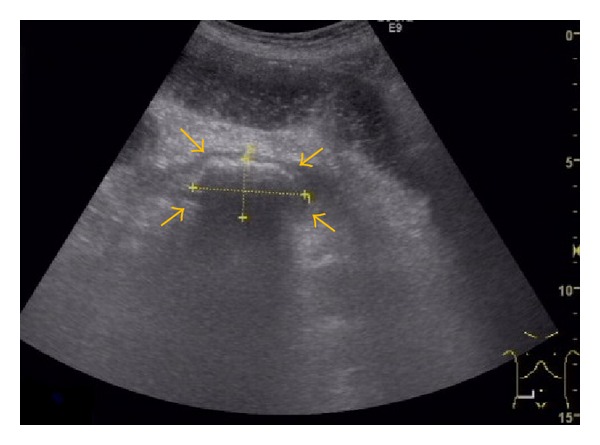
Abdominal ultrasound image on hospital day 59 showing a hyperechoic solid space-occupying lesion (45 × 23 mm) with acoustic shadowing (arrows) in the ileocecal region.

**Figure 5:**
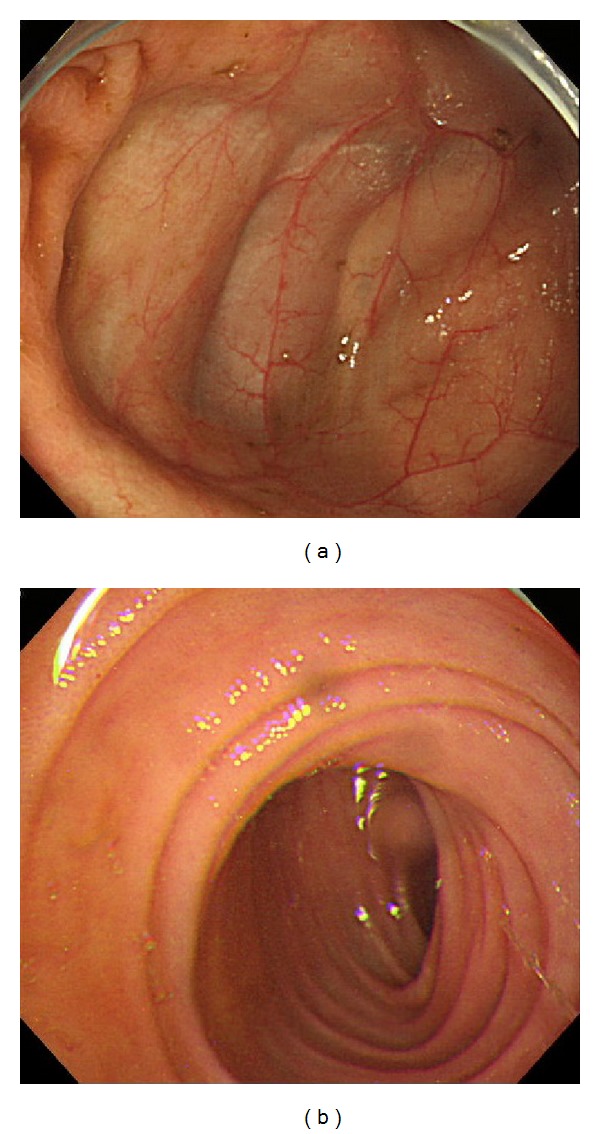
Colonoscopy on hospital day 66 showing (a) the ileocecal region and (b) ileum. No stone was observed during observation of the small intestine up to 7 cm proximal to the terminal ileum.

**Figure 6:**
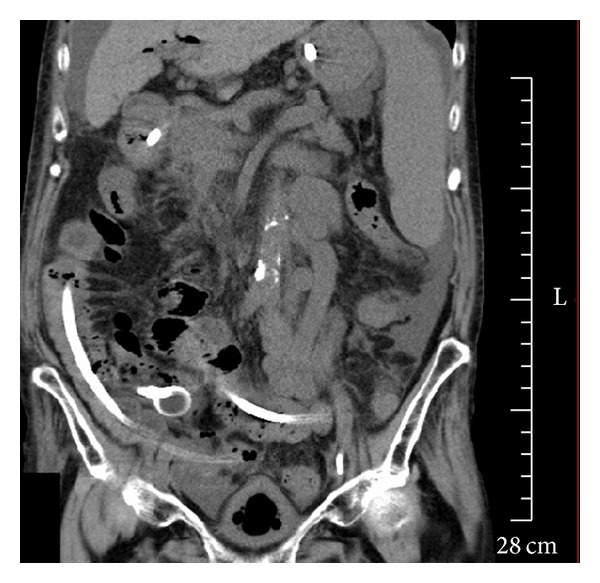
Abdominal CT scan performed immediately after colonoscopy showing the absence of a stone and improved intestinal distension.

The patient developed sudden abdominal pain and nausea on hospital day 58 and was diagnosed as having ileus on the following day based on the CT findings of abdominal distension and the accumulation of intestinal fluid. CT also revealed that the stone, which had been passed into the duodenum in the previous ERCP, was incarcerated in the ileocecal region (Figure 3), and this was verified by the ultrasonography findings of a 45 × 23-mm stone with acoustic shadowing at the same location (Figure 4). Based on these findings, we diagnosed the patient as having gallstone ileus caused by an incarcerated gallstone in the ileocecal region. First, an ileus tube (ileus tube 16 Fr, 5.3 mm outer diameter, 300 cm in length, Fuji Systems Co., Tokyo, Japan) was inserted nasally to reduce the pressure inside the intestine, and doing so improved abdominal pain and nausea. On hospital day 66, to remove the incarcerated stone, we inserted a colonoscope to approximately 7 cm from the terminal ileus; however, no stone was observed (Figure 5). The disappearance of the stone was verified by CT performed immediately after colonoscopy, suggesting that the stone had been evacuated naturally via the transanal route (Figure 6). Ileus did not relapse.

## 3. Discussion

First reported by Erasmus et al. in 1654, gallstone ileus is a rare disease [1] accounting for 1–3% of all cases of mechanical obstruction of the small intestine. The incidence of gallstone ileus is high in the elderly, accounting for 25% of cases of nonstrangulation small-intestinal obstruction in individuals >65 years old, with a mortality rate of 15–18% [2]. Many gallstones pass into the intestinal track through the fistula that develops due to cholecystitis, and only 0.8% of gallstone ileus cases occur naturally via the bile duct [3]. The development of gallstone ileus after ERCP, as in this study, was first reported by Halter et al. in 1981 [4], and this study was followed by 11 reports of gallstone ileus preceded by ES [1, 4–13]. Although the Vater papilla is edematous after ES, once edema has subsided, large stones can be removed. Stones incarcerated in the intestinal tract are reportedly larger than 25 mm, but Oskam et al. stated that stones about 20–30 mm in size can also naturally pass into the duodenum after ES [5]. When we first performed endoscopic stone removal in the present case, the Vater papilla was located in the diverticulum in the third part of the duodenum, forcing us to use the push method and operate manually without visual confirmation of the bile duct opening. Assuming that stone removal would be difficult, we instead placed a plastic stent to prevent cholangitis and performed regular stent replacement. The factor which contributed to the stone removal by the balloon catheter is thought to be the nonedematous Vater papilla and consequently the wider opening to the bile duct because a long time had passed since the last ES.

In addition to stone size, patient characteristics are important factors in gallstone ileus. More than 60% of gallstones are said to be incarcerated in the ileum [2], largely because of the ileum's narrow diameter, strong curves, and infrequent peristalsis. According to Pittman et al., advanced age and severe comorbidities are risk factors for gallstone ileus. It is particularly important to pay attention to patients with possible stenosis of the small intestine due to a history of radiotherapy for cervical cancer or ileocecal resection for Crohn's disease [6]. The present patient had no history of radiotherapy or open abdominal surgery. However, accumulation of ascites due to hepatitis B virus-related cirrhosis and declined physical activity due to a lumbar compression fracture appeared to have reduced the intestinal peristaltic activity in the present patient, which in turn caused the incarceration of the gallstone in the ileocecal region.

Diagnostic imaging is essential for the diagnosis of gallstone ileus. Even though 77.8% of air-fluid levels and 88.9% of abdominal distension cases are diagnosed on plain abdominal radiography, only 37% of pneumobilia cases, a condition unique to gallstone ileus, and 33% of ectopic gallstone cases [14] are diagnosed, thereby making the diagnosis of gallstone ileus based solely on plain radiographic images difficult. At present, CT is considered the most useful tool for diagnosing gallstone ileus. In a prospective study conducted by Yu et al., the diagnostic performance of the criteria used in enhanced CT imaging of small intestinal obstruction, ectopic gallstones, and abnormalities in the gallbladder (pneumobilia, inflamed wall, and thickened wall) had a sensitivity of 93%, specificity of 100%, and accuracy of 99% [15].

In principle, gallstone ileus requires treatment intervention since the disease condition improves naturally in only 4–8% of cases. The goal of intervention is to cure ileus by removing stones from the intestinal tract, which is achieved mostly by surgical intervention, but the use of an endoscope [16–27] and extracorporeal shock wave lithotripsy [28–31] has also been reported. It remains controversial whether to perform stone removal surgery alone or with fistula closure and cholecystectomy. While some suggest that the latter should be performed to prevent the recurrence of cholecystitis and gallstone ileus [32–34], others insist that the possibility of recurrence is low once the stone is removed and that the fistula will close in due course [35, 36]. The present patient was in poor general health and had Child-Pugh grade C cirrhosis as the underlying disease, which led to the indication of endoscopic procedures but not surgical intervention. As a result, the gallstone was evacuated naturally via the transanal route, presumably because of the lack of stenosis in the small intestine and the improvement of intestinal peristalsis after inserting an ileus tube to reduce intestinal pressure on the day of onset.

## 4. Conclusions

We recently encountered a rare case of gallstone ileus that developed after endoscopic stone removal. The present findings suggest that when endoscopically removing huge stones, surgeons should pay attention to the risk of gastrointestinal obstruction, as well as stone size, and follow patients carefully.

## Figures and Tables

**Table 1:** Blood examination results.

Hematology		Biochemistry	
WBC	10500/*μ*L	TP	4.8 g/dL
RBC	255 × 10^4^/*μ*L	Alb	2.5 g/dL
Hgb	7.3 g/dL	T-bil	6.8 mg/dL
Ht	23.2%	D-bil	6.1 mg/dL
MCV	91.0 fl	AST	58 U/L
MCH	28.6 pg	ALT	22 U/L
MCHC	31.5%	LDH	291 U/L
PLT	3.2 × 10^4^/*μ*L	ALP	406 U/L
		GGT	60 U/L
Coagulation test		BUN	67 mg/dL
PT	37%	Cr	1.87 mg/dL
PT-INR	1.90	Na	132 mEq/L
		K	4.9 mEq/L
		Cl	108 mEq/L
		CRP	14.9 mg/dL

WBC, white blood cell count; RBC, red blood cell count; Hgb, hemoglobin; Ht, hematocrit; MCV, mean corpuscular volume; MCH, mean corpuscular hemoglobin; MCHC, mean corpuscular hemoglobin concentration; PLT, platelet; PT, prothrombin time; PT-INR, prothrombin time international normalized ratio; TP, total protein; Alb, albumin; T-bil, total bilirubin; D-bil, direct bilirubin; AST, aspartate aminotransferase; ALT, alanine transferase; LDH, lactate dehydrogenase; ALP, alkaline phosphatase; GGT, gamma-glutamyltranspeptidase; BUN, blood urea nitrogen; Cr, creatinine; CRP, C-reactive protein.
